# Tissue Integration of Calcium Phosphate Compound after Subchondroplasty: 4-Year Follow-Up in a 76-Year-Old Female Patient

**DOI:** 10.3390/bioengineering10020208

**Published:** 2023-02-04

**Authors:** Samo K. Fokter, Matevž Kuhta, Marko Hojnik, Živa Ledinek, Rok Kostanjšek

**Affiliations:** 1 Department of Orthopaedics, University Medical Centre, 2000 Maribor, Slovenia; 2 Department of Pathology, University Medical Centre, 2000 Maribor, Slovenia; 3 Department of Biology, Biotechnical Faculty, University of Ljubljana, 1000 Ljubljana, Slovenia

**Keywords:** subchondroplasty, osteoarthritis, knee, micro-CT, calcium phosphate compound, arthroplasty, bone tissue regeneration, biomedical device, implant interface, orthopedic implant

## Abstract

Subchondroplasty is a new minimally invasive surgical technique developed to treat bone marrow lesions (BML) and early osteoarthritis (OA). During the procedure, engineered calcium phosphate compound (CPC) is injected. It is claimed by the manufacturer that during the healing process, the CPC is replaced with new bone. The purpose of this study was to verify the replacement of CPC with new bone after subchondroplasty for the first time in humans. A 76-year old woman was referred for resistant medial knee pain. Standing radiographs showed varus knee OA and magnetic resonance imaging (MRI) revealed BML. She was treated with subchondroplasty of medial femoral condyle. Excellent relief of pain was achieved after procedure. Afterwards, the pain worsened, the radiographs confirmed the OA progression and the patient was treated with a total knee arthroplasty (TKA) 4 years after primary procedure. The resected bone was examined histologically and with micro-computed tomography (CT). Histologically, bone trabeculae of subcortical bone were embedded in the amorphous mass. However, no signs of CPC resorption and/or bone replacement have been found with micro-CT. In short term, excellent pain relief could be expected after the subchondroplasty procedure. However, there was no replacement of CPC with bone and the technique probably did not influence the natural process of knee OA.

## 1. Introduction

Subchondroplasty is a new fluoroscopic-guided minimally invasive surgical technique developed to treat subchondral bone defects, bone marrow lesions (BML) and early osteoarthritis (OA) [1,2,3,4,5]. In older adults OA is the leading cause of disability and represents considerable costs to the society. The incidence of OA is influenced by many factors, such as work, sports participation, musculoskeletal injuries, obesity, and gender [6]. Hip and knee OA was ranked as the 11th highest contributor to global disability and 38th highest in disability-adjusted life years among 291 conditions [7]. The prevalence of hip and knee OA was higher in females than in males and has shown no discernible change in recent decades. With the high likelihood that the real burden of OA has been underestimated because of methodological issues, the global age-standardized prevalence of hip OA was 0.85% (95% uncertainty interval (UI) 0.74% to 1.02%). In this way, it was almost 4.5 times lower than the prevalence of knee OA, which was 3.8% (95% UI 3.6% to 4.1%) [7]. Under the line, it was estimated that knee OA constitutes 83% of the global disease burden for OA [8]. With the aging and increasing obesity of the global population, the demand for a large increase for health services to treat OA is expected to grow exponentially over the coming decades. This is particularly true for knee OA. End-stage knee OA is commonly treated with total knee arthroplasty (TKA), which represents a major and costly surgical procedure. Some epidemiological data are suggesting a 673% increase of the TKAs in the United States by 2030, representing 3.48 million procedures annually [9]. Less invasive treatments of chondral lesions representing early stages of OA with prolonged therapeutic effect would therefore be much appreciated [10]. 

Traditionally, OA was graded with radiological assessment. Kellgren and Lawrence developed a useful 4-grade radiograph-based classification system of OA back in 1957 which is still widely used [11]. According to Kellgren and Lawrence system, Grade 1 demonstrates doubtful narrowing of the joint space with possible osteophyte formation, Grade 2 demonstrates possible narrowing of the joint space with definite osteophyte formation, Grade 3 demonstrates definite narrowing of joint space, moderate osteophyte formation, some sclerosis, and possible deformity of bony ends, and Grade 4 demonstrates large osteophyte formation, severe narrowing of the joint space with marked sclerosis, and definite deformity of bone ends. Magnetic resonance imaging (MRI) nowadays represents the standard clinical utility in detecting anatomical location and grade of cartilage lesions. Unfortunately, only approximately 30% of knee MRI showed an adequate cartilage status in all anatomical locations [12]. On the other hand, degenerative lesions around the knee are the common cause of secondary bone marrow edema (BME) or BML easily detected by MRI [13,14,15,16]. Many studies have shown that BME corresponds to knee pain in OA [17,18,19]. Despite the etiology of BML is multimodal, it represents a universal bone response to injury [20,21,22,23,24]. Studies on non-traumatic necrosis of the femoral head, advanced and rapidly destructive hip OA, and transient osteoporosis of the hip have shown that these MRI detected bone marrow alterations correspond histologically with true edema, trabecular necrosis, and overstressed subchondral bone [25,26,27,28,29].

The subchondroplasty technique uses an orthobiologic that mimics the strength of subchondral bone to support bone repair [30]. The idea to use a synthetic bone graft instead of autologous bone graft was developed from the experience gained by the orthopedic community from tumor, fracture, and BME treatment [31,32,33,34,35]. During the subchondroplasty procedure, engineered calcium phosphate compound (CPC) in a flowable form is injected under fluoroscopic guidance as bone void filler through a cannulated needle into the space between the trabeculae of subchondral cancellous bone in the area of a MRI detected BML. Consequently, CPC crystallizes in an endothermic reaction and forms nanocrystalline, macroporous scaffold in the bone which is believed to be replaced by bone cells over time [30]. In a goat model of experimental tibial plateau fractures augmented either with calcium phosphate (CaP) cement or autologous bone graft, the research have namely shown that augmentation with CaP cement prevented subsidence of fracture fragment, maintained articular congruency as the fracture healed, was rapidly resorbed, and the volume fraction of the CaP cement was decreased to 4% at 6 months [36]. Consequently, it is claimed by the manufacturer of a commercially available subchondroplasty system that during the healing and remodeling process, the CPC is replaced with new subchondral bone also in humans. In this way, the potential for restoring biomechanical integrity of the articular cartilage and the whole joint would be established in a less invasive way thus reducing morbidity for the patients and the costs for the society [37]. The aim of this study was to verify the replacement of CPC with new bone after subchondroplasty in an illustrative case. 

## 2. Materials and Methods

**Figure 1:**
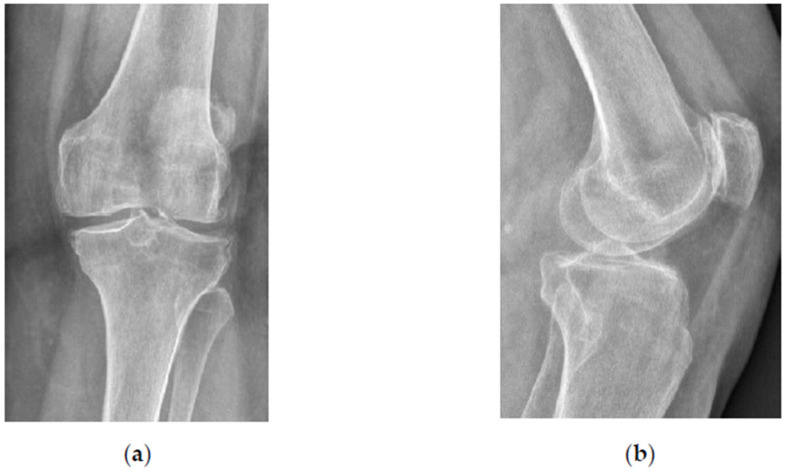
Standing X-ray images of the left knee. (**a**) Anteroposterior view; (**b**) lateral view.

**Figure 2:**
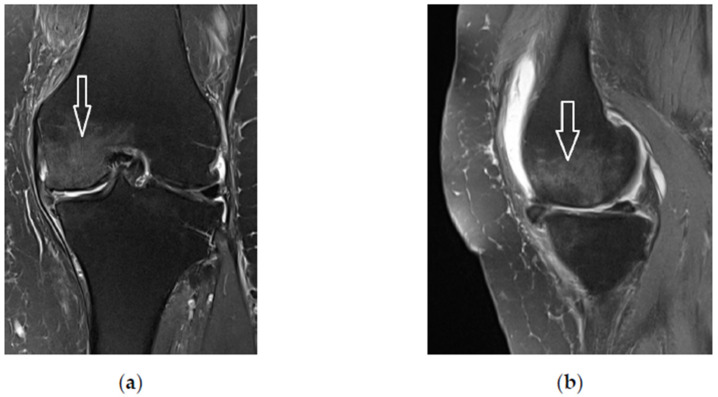
T2-weightened magnetic resonance imaging (MRI) of the left knee. Note bone marrow edema of the medial femoral condyle (arrows). (**a**) Coronal plane; (**b**) sagittal plane.

A 76-year old woman was referred for progressive left knee pain lasting for over 1 year and the pain was resistant to standard nonoperative treatment. The patient scored her pain 8 on the visual analogue scale (VAS). Standing radiographs showed varus knee OA of Kellgren-Lavrence grade 2 (Figure 1). Magnetic resonance imaging (MRI) showed an alteration of bone marrow signal intensity on fluid-sensitive sequences (Figure 2).

**Figure 3:**
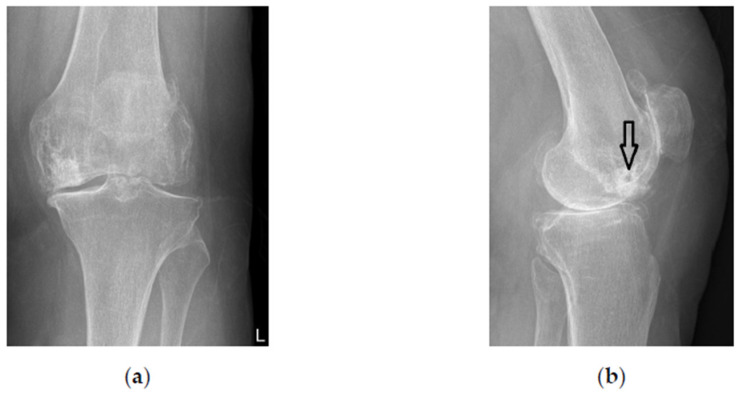
Standing X-rays of the left knee 4 years after subchondroplasty. Note calcium phosphate compound (CPC) filling the subchondral bone of the medial femoral condyle. (**a**) Anteroposterior view; (**b**) lateral view. The hole from the delivering cannula is clearly visible (arrow).

She refused total knee arthroplasty (TKA) and was treated with subchondroplasty of her left knee (MK). During the procedure, 3 ccm of CPC (AccuFill^®^, Zimmer Knee Creations, Exton, PA, USA) bone substitute material (BSM) was prepared in a special container (AccuMix^®^ Mixing System, Zimmer Knee Creations) and delivered to the medial femoral condyle under intraoperative radiographic guidance through an 11-gauge jamshidi-type perforated needle (AccuPort^®^ Cannula, Zimmer Knee Creations). Excellent relief of pain was achieved immediately after procedure (VAS score 2 at 3 weeks) and lasted for 3 years. Afterwards, the pain has worsened to VAS score 7, the radiographs confirmed the OA progression to Kellgren-Lawrence grade 3 (Figure 3) and the patient was scheduled for a TKA 4 years after the primary procedure. 

**Figure 4:**
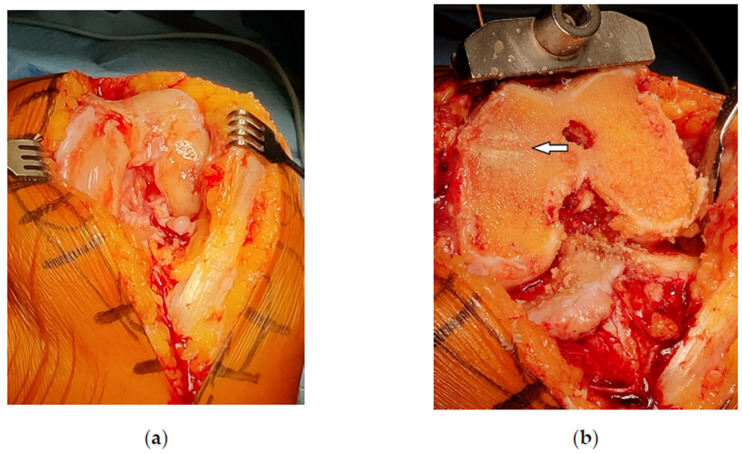
Intraoperative photographs of the left knee during total knee arthroplasty (TKA). (**a**) Synovial proliferation in the intercondylar notch; (**b**) amorphous material in the medial femoral condyle. Note the central groove from the delivering cannula (arrow).

**Figure 5:**
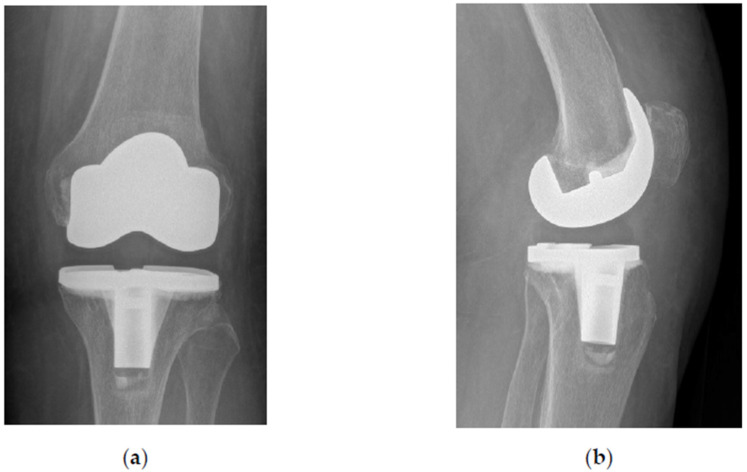
Standard X-rays of the left knee after total knee arthroplasty (TKA). (**a**) Anteroposterior view; (**b**) lateral view.

During the TKA procedure abundant synovial proliferation was noted and the cancellous bone of the medial femoral condyle was filled with amorphous material (Figure 4). The standard posterior stabilized total knee prosthesis (NexGen LPS-Flex, Zimmer Biomet, Warsaw, IN, USA) was inserted uneventfully (SKF). The patient followed the fast-track rehabilitation protocol and was discharged from the hospital 3 days after admission (Figure 5). 

The soft tissues and the resected bone of the medial femoral condyle were sent for histologic examination. Brown to grey tissue fragments of soft to firm consistency, measuring 4 to 15 mm, were formalin-fixed and only firm fragments were additionally decalcified in trichloroacetic acid (Carlo Erba Reagents GmbH, Cornaredo, Italy). All tissue underwent routine tissue processing and was paraffin-embedded. Out of each paraffin-embedded tissue block, 3–5 µm thick paraffin sections were cut and placed on glass slide (Superfrost, Waldemar Knittel Glasbearbeitungs GmbH, Braunschweig, Germany). Hematoxylin and eosine (HE) staining was performed on automated Ventana HE 600 system (Ventana Medical Systems, Roche, Basel, Switzerland). The stained tissue sections were evaluated by an experienced board-certified pathologist (MH). Microphotographs of tissue slides were taken with Aperio ScanScope CS (Aperio, Leica Biosystems, Nußloch, Germany) with 20× scanning magnification and exported from Aperio ImageScope (version: 12.4.6.5003).

A microphotograph of the resected medial femoral condyle was taken. Additionally, the amorphous material filling the cancellous bone was checked with Keyence VHX 7000 stereomicroscope (Keyence Corporation, Osaka, Japan) with up to 6000 times magnification. 

Finally, micro-computed tomography (micro-CT) was performed to evaluate the process of CPC replacement with new bone. The resected distal femoral condyle of the left knee was scanned on Neoscan N80 microtomograph (Neoscan, Mechelen, Belgium) at 92 kV, 173 µA, with Cu 0.25 mm filter at 10 µm resolution, with a rotation step of 0, 3° for 180° and averaging set to 2. Virtual cross sections were reconstructed from three sets 660 projection images covering the full height of the sample by Neoscan 80 software (version 2.2.4) (Neoscan, Belgium). A total of 4610 sections were imported into Dragonfly software (version 2021.1.0.977) (ORS Inc., Mississauga, Ontario, Canada) for 3D reconstruction and segmentation analysis.

## 3. Results

### 3.1. Surgical Examination

Before TKA insertion, the patient’s left knee was clinically swollen, euthermic, and axially deformed in varus. The range of motion (ROM) was reduced (flexion/neutral/extension) to 100°/5°/0° (normally 135°/0°/5°). Medial joint line was very painful on palpation. With the patient under spinal anesthesia, a tourniquet was inflated and medial arthrotomy was performed. The joint was carefully examined for possible CPC particles, which were not found. Macroscopically, there were no signs of articular cartilage regeneration over the bare bone surface of the medial femoral condyle. With the help of an intramedullary guidance, the distal femoral resection was performed with the oscillating saw perpendicular to mechanical femoral axis. The medial femoral condyle was found sclerotic and resistance was felt to sawing. The cancellous bone of the remaining proximal part of the medial femoral condyle was filled with CPC which was impossible to separate from the remaining bone trabeculae without the danger of creating a large cavity thus compromising the bearing capacity for the femoral component of the TKA.

### 3.2. Histologic Examination

**Figure 6:**
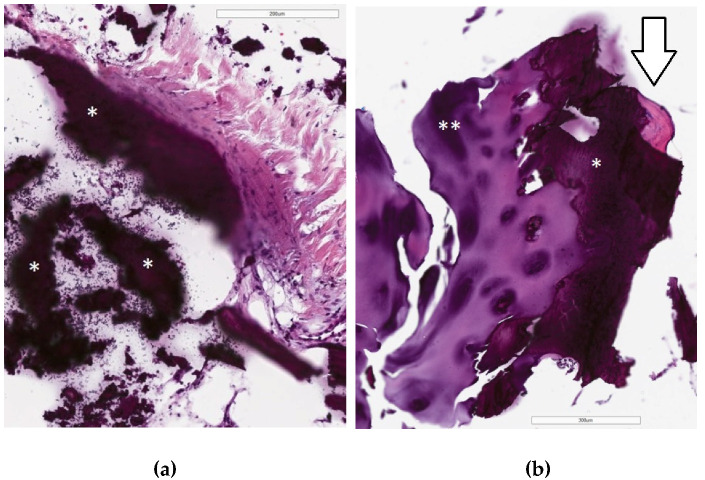
Photomicrograph of HE stained tissue slides from resected distal femoral condyle of the left knee. (**a**) Abundant amount of amorphous basophilic material (*) is seen adjacent to the articular tissue (150× magnification); (**b**) amorphous material (CPC) (*) is present in between the cartilage (**) and subcortical bone (arrow) with no sign of resorption (200× magnification).

Histologically, fragments of bone trabeculae of subcortical bone and cartilage were present in contact with the amorphous basophilic mass with some black pigment, resembling calcium phosphate. No signs of CPC resorption and bone necrosis were seen. Synovia showed moderate hyperplasia of lining layer, underlining hypocellular fibrosis with focal minimal lymphocytic infiltrate and focal capillary hyperplasia, which are features of chronic synovitis. Adjacent bone, cartilage, and soft tissue were morphologically normal (Figure 6).

### 3.3. Microphotography and Stereomicroscopic Examination

**Figure 7:**
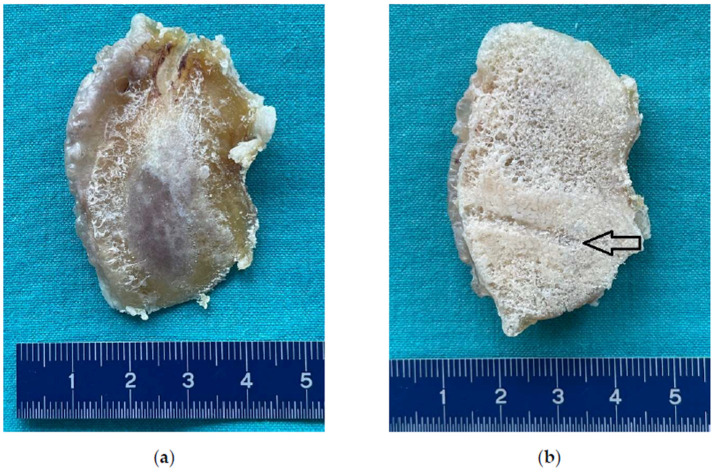
Photomicrograph of the resected distal femoral condyle of the left knee. (**a**) Caudal view; (**b**) cranial view. Note the central groove from the delivering cannula (arrow).

**Figure 8:**
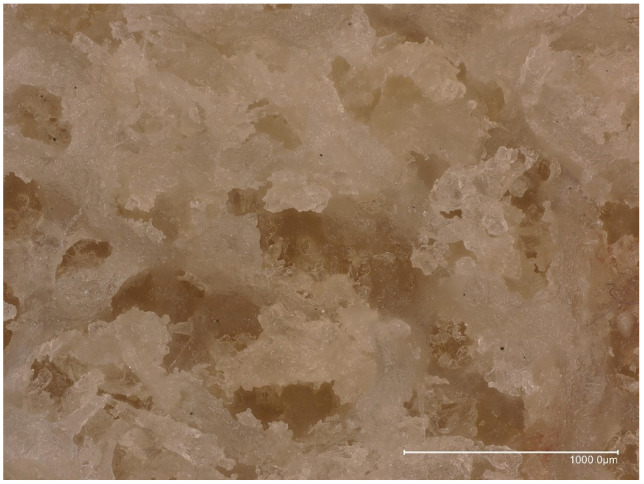
Stereomicroscopic photograph of the CPC material filling the cancellous bone of the resected medial femoral condyle.

Microphotography of the resected distal femoral condyle revealed cancellous bone filled with cement material spreading centrifugally from the still visible channel hollowed out by the subchondroplasty needle at the time of primary procedure. No signs of CPC resorption, bone remodeling, and/or slowing down the natural progression of the OA were present (Figure 7). Stereomicroscopic examination revealed amorphous CPC material embedding cancellous bone trabeculae without any signs of bone ingrowth (Figure 8). 

### 3.4. Micro-CT Examination

**Figure 9:**
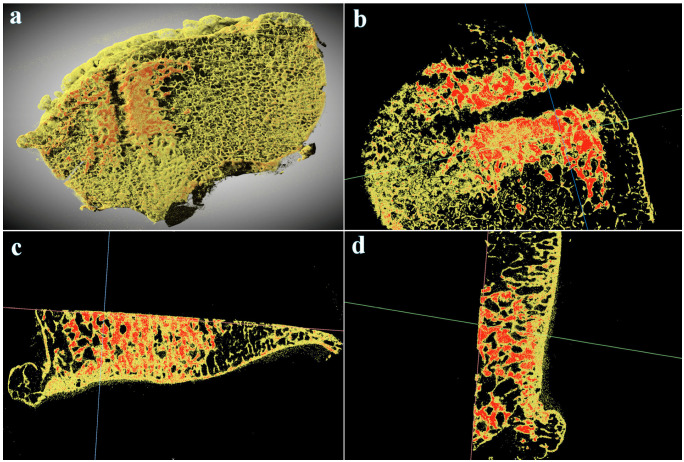
Micro-CT analysis of the resected distal femoral condyle of the left knee. (**a**) 3D reconstruction of the condyle in cranial view showing denser CPC material (in red) surrounding the central groove of the delivering cannula. (**b–d**) Orthogonal views of the same area showing denser amorphous CPC (in red) filling spaces between a les denser trabeculae (in yellow) with unchanged architecture.

Micro-CT analysis of the resected femoral condyle enabled distinction between a denser CPC from a les dese bone tissue. Segmentation analysis based on material density revealed amorphous CPC filling the intratrabecular spaces of subchondral bone surrounding the channel drilled out during subchondroplasty (Figure 9). Detection of CPC surrounding a trabelcular bone arranged in a distinct architecture strongly indicates absence of bone remodeling, ingrowth after CPC treatment, or CPC resorption.

## 4. Discussion

To the best of our knowledge, the present study is the first to take a closer look at the bone remodeling process after subchondroplasty in humans.

Several studies confirmed that patients experience short-term improvements in pain and functional outcomes following the subchondroplasty procedure for painful subchondral BMLs. Chua et al. reported on 12 patients with symptomatic BML in the knee, including knees with early OA [38]. The patients were evaluated with standard radiographs and MRI was utilized to confirm and localize the BML. The patients were treated with subchondroplasty and followed for clinical efficacy. At the 1-year follow-up, the VAS pain scores, Western Ontario and McMaster Universities Osteoarthritis Index (WOMAC) scores, and Knee Injury and Arthritis Outcome Scores (KOOS) improved significantly. Our experience with subchondroplasty in this particular case was also very promising on the short-term.

Nairn et al. performed a systematic review in 2020 to summarize the patient-related outcomes of subchondroplasty [39]. The authors concluded that existing low-quality studies confirmed patients with BMLs to benefit from subchondroplasty procedure through reduction in pain and improvement in function. Moreover, low conversion rate to THA (12.5–30%) was noted in the short-to-medium term follow-up suggesting that subchondroplasty may postpone more invasive procedures in patients with BMLs. However, the authors acknowledge that these promising initial findings should be further evaluated with high-quality comparative studies with long-term follow-up. The results of the aforementioned review were recently confirmed by Krebs et al., who followed 12 patients after knee arthroscopy with adjunctive subchondroplasty performed for knee pain associated with BMLs for 3 years on average (range, 12–51 months) [40]. Significant reduction of mean preoperative VAS scores compared to six-week postoperative VAS scores and final postoperative VAS scores were noted. Moreover, only 2 patients (16.7%) required conversion to TKA at 36-month follow-up. We agree with Nairn et al. that most of the patients were followed by the same surgeon who performed their initial subchondroplasty and that the indications for TKA are very physician-dependent, thus the rates of converting to THA might be biased. Our patient was offered the free choice of treatment through the whole medical process. It is not insignificant to mention that the surgical management was totally free of costs for the patient. Moreover, it has been clearly shown that clinically significant improvements in pain and function occur after placebo intra-articular saline injections in knee OA [41]. Unfortunately, no placebo-controlled subchondroplasty study exists in the medical literature [42,43,44,45].

It has also been shown in a systematic review that intraosseous injections of platelet-rich plasma (PRP) are comparatively safer and effective to subchondroplasty in knee OA [46]. Combining subchondroplasty with PRP or bone marrow aspirate concentrate (BMC) has been tested in a validated preclinical canine model for post-traumatic cartilage and subchondral BML induced with arthroscopic impact injury [37]. Three months after the mentioned injury, one knee in each of 24 dogs was randomly assigned to treatment with subchondroplasty and the other knee to subchondroplasty and PRP, subchondroplasty and BMC, or sham injection (control). The knees were evaluated using radiography, arthroscopy, and MRI and the dogs were euthanatized at 1 year after treatment for histologic assessments. At 1 year follow-up, arthroscopic scores revealed no statistically significant differences between the treatment groups. However, knees treated with subchondroplasty and PRP and subchondroplasty and BMC had significantly better MRI grades than subchondroplasty-only treated knees and controls. Histologically, the authors were also able to show that in the subchondroplasty-treated medial femoral condyles the majority of CPC was resorbed, especially in the PRP-augmented and BMC-augmented groups. The study was sponsored by Zimmer-Biomet, Inc. [37]. While the results of treatment in animal models are not easily compared to the results of treatment in humans, we were certainly not able to retrieve a comparative amount of medial femoral condyle in our patient for the histological analysis. However, no signs of CPC resorption were evident in the medial femoral condyle of the patient presented in this study.

Our results with micro-CT analysis of the cancellous subchondral bone 4 years after subchondroplasty have proven the adequate injectability of used CPC. This bone substitute material (BSM) characteristic has been tested with some other commercially available products, including classic poly-methyl-methacrylate (PMMA) bone cement [47]. In a study on a simulated trabecular model confirmed with cadaveric samples, the authors examined 8 BSMs regarding their function in small microarchitecture areas. The micro-CT analysis has shown that only 2 nanocrystalline CaPO_4_ preparations (AccuFill^®^, Zimmer, Inc. and StrucSure™, Smith & Nephew, Inc. Memphis, TN, USA) beside 1 PMMA tested (Simplex™, Stryker^®^) demonstrated a notable volume injected into the foam block and interdigitated into the architecture. The authors concluded that not all as “injectable” described BSM materials function well in a small microarchitecture in comparison to the large void areas [47].

On the contrary, our results with micro-CT analysis are in sharp contrast with the results of a study performed on an impact-induced subchondral BML in a validated preclinical canine model [30]. Three months after arthroscopic impact injury of both medial femoral condyles with a custom-designed spring-driven impactor with a 8-mm diameter tip (40 N at a rate of 100 mm/s), 32 knees of 16 hounds were randomly assigned to fluoroscopically guided percutaneous subchondroplasty (AccuFill^®^, Zimmer, Inc.) or sham injection (control). Dogs were assessed at regular intervals 24 months after treatment using functional assessments, radiographic evaluation, arthroscopy, and MRI. After euthanatization, micro-CT and histopathology were performed. At 1- and 2-year posttreatment, range of motion was significantly better in subchondroplasty-treated knees, while radiographic, arthroscopic, MRI, and histologic findings matched the functional assessment. With the micro-CT, the authors demonstrated trabecular thickness and bone volume fraction to be higher in the subchondroplasty group compared with the control group. On the other hand, trabecular spacing was lover in the subchondroplasty group compared with the control group. The authors were also able to show that the majority of the injected CPC material was resorbed by the 2-year endpoint with the remaining material being proximal to the load bearing portion of the condyles. The study was sponsored by Zimmer, Inc. [30]. As noted above, the results of treatment in animal models are not easily compared to the results of treatment in humans. Moreover, our study incorporated no control. However, with the micro-CT analysis we were certainly not able to detect any signs of CPC resorption of the medial femoral condyle of the patient presented in our study.

We acknowledge certain limitations in this study. First, it is clear that with only one case examined, it is not possible to draw solid conclusions about the effect of the independent variable. Replicates determined by statistical analysis are needed to prove or disapprove a hypothesis. We are planning to thoroughly follow all patients treated with subchondroplasty at our institution to get more data on the bone integration of the CPC being used as bone filler and in pain alleviation associated with subchondral bone lesions in OA. Secondly, special staining and immunohistochemical analysis were not performed in our study. However, additional special staning or the use of immunohistochemical methods to further depict the ultrastructure of the analysed tissue would not influence the final diagnosis in any way. Standard hematoxylin and eosin staning enables an accurate description of the morphology which was necessary for the diagnosis in this case. Immunohistochemical analysis can only be done on living cells and it would be exessive to use in this specific case. Special stains (i.e., Masson’s Trichrome) could be used to differentiate between cartilage and bone but would be of little help to differentiate the amorphus material from the surrounding tissue and would therfore be of little value as well. Third, since there was no control in our study, we did not include measurements of trabecular thickness, trabecular spacing, and bone volume fraction in our micro-CT analysis.

## 5. Conclusions

In short term, excellent pain relief could be expected after the subchondroplasty procedure with the CPC in carefully selected patients with BML accompanying early OA. However, the replacement of CPC with bone in humans might be much slower than previously expected on the basis of animal experiments. Moreover, it seems that the technique does not influence the natural progression of knee OA. Further prospective randomized studies on larger number of patients are needed to confirm our case-based results.

## Data Availability

Not applicable.
